# Liver Cancer: Artificial Intelligence (AI)-Based Integrated Therapeutic Approaches

**DOI:** 10.3390/bioengineering12080837

**Published:** 2025-08-01

**Authors:** Mythileeswari Lakshmikanthan, Sakthivel Muthu, John T. D. Caleb, Yuvaraj Maria Francis, Indra Neel Pulidindi

**Affiliations:** 1 Department of Biotechnology, University of Madras, Guindy Campus, Chennai 600025, India; mythileeswari@gmail.com; 2 Department of Dermatology, Saveetha Medical College and Hospital (SMCH), Saveetha Institute of Medical and Technical Sciences (SIMATS), Thandalam, Chennai 602105, India; saktthivel@gmail.com; 3 Department of Anatomy, Saveetha Medical College & Hospital, Saveetha Institute of Medical and Technical Sciences Deemed University, Chennai 602105, India; araneae.in@gmail.com (J.T.D.C.); sujinalways@gmail.com (Y.M.F.); 4 Department of ENT, Saveetha Medical College & Hospital, Saveetha Institute of Medical and Technical Sciences Deemed University, Chennai 602105, India

The advent of artificial intelligence and machine leaning techniques has revolutionized the diagnosis and therapy of diseases such as cancer [1,2,3]. A Web of Science search with the keywords “artificial intelligence and diagnosis and and therapy” yields 3034 results as of 31 July 2025, indicating the amount of research of this area. Liver cancer, predominantly hepatocellular carcinoma (HCC), stands as the sixth most commonly diagnosed malignancy and the third leading cause of cancer-related deaths globally [4]. Its multifactorial origin encompasses chronic hepatitis B and C virus infections, alcohol-induced liver cirrhosis, non-alcoholic fatty liver disease (NAFLD), aflatoxin B1 exposure, and metabolic syndromes. The aggressive nature of HCC and its typically late-stage diagnosis result in poor prognosis and limited therapeutic success [5]. In addition to its silent clinical progression, the heterogeneity of tumor biology presents a significant challenge in effective disease management [6,7]. Geographic and ethnic variations further influence the incidence and origin of HCC, with developing regions experiencing a disproportionately higher disease burden due to inadequate screening and vaccination programs. Recent advances in molecular biology and genomics have elucidated the key oncogenic signaling pathways involved in liver carcinogenesis. They include Wnt/β-catenin, PI3K/Akt/mTOR, MAPK/ERK, and TGF-β pathways. These discoveries have paved the way for the development of targeted therapies, including multi-kinase inhibitors like sorafenib and lenvatinib, which offer modest survival benefits (Figure 1). However, therapeutic resistance and adverse side effects remain major hurdles [8,9], which are surmountable with the judicious use of AI- and ML-based methods.

The emergence of immunotherapy, and particularly the use of immune checkpoint inhibitors targeting programmed cell death protein 1 (PD-1), its ligand PD-L1, and cytotoxic T-lymphocyte-associated antigen 4 (CTLA-4), has demonstrated significant promise in enhancing antitumor immune responses in patients with hepatocellular carcinoma (HCC). These agents work by restoring T-cell activation and enabling the immune system to recognize and eliminate tumor cells more effectively [10,11]. While monotherapy with checkpoint inhibitors has yielded encouraging results in a subset of patients, response rates remain variable [12].

Immunotherapy combined with anti-angiogenic agents such as bevacizumab normalizes tumor vasculature promoting immune cell infiltration, as does immunotherapy combined with locoregional treatments like transarterial chemoembolization (TACE) and radiofrequency ablation (RFA). These techniques induce immunogenic cell death and enhance immune priming. Such multimodal approaches are under active clinical investigation, aiming to improve overall survival, progression-free survival, and long-term tumor control in advanced-stage HCC patients [13,14]. Despite these advancements, early detection remains critical. Current diagnostic tools, including imaging techniques and serum biomarkers like alpha-fetoprotein (AFP), lack sufficient sensitivity and specificity for detecting early-stage HCC. Thus, novel biomarkers and liquid biopsy approaches are being explored to enhance diagnostic precision and monitor treatment response [15,16].

Prevention strategies, particularly vaccination against hepatitis B virus and antiviral therapies for hepatitis C, have significantly reduced the incidence of HCC in high-risk populations. Nevertheless, the global burden of liver cancer is projected to rise due to the increasing prevalence of obesity and NAFLD-related hepatic disorders [17,18]. In conclusion, liver cancer remains a formidable threat to life. A multidisciplinary approach based on artificial intelligence (AI), machine learning (ML), and nanotechnology, integrating prevention, early diagnosis, personalized effective therapy, and continuous care, is essential to improve clinical outcomes [19]. Future research should focus on uncovering novel molecular targets, optimizing therapeutic combinations, and refining diagnostic methodologies to combat this life-threatening malignancy effectively with the use of combinatorial biochemistry and high-throughput screening techniques [20,21,22,23,24,25,26,27,28,29,30,31,32,33,34,35,36,37].

## Figures and Tables

**Figure 1:**
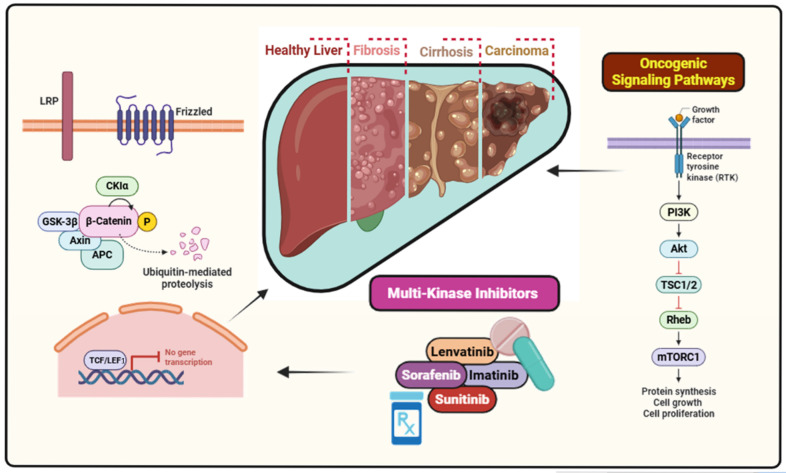
Illustration of liver cancer progression and targeted inhibition of Wnt/β-catenin and PI3K/Akt/mTOR pathways by multi-kinase inhibitors.

## Abbreviations

The following abbreviations are used in this manuscript:

AFP	Alpha-fetoprotein
AI	Artificial intelligence
APC	Adenomatous Polyposis Coli gene
CTLA-4	Cytotoxic T-lymphocyte-associated antigen 4
HCC	Hepatocellular carcinoma
LEF-1	Lymphoid enhancer factor—1
LRP	Low-density lipoprotein receptor-related protein
mTOR	Mammalian target of rapamycin
NAFLD	Non-alcoholic fatty liver disease
PD-1	Programmed cell death protein 1
RFA	Radiofrequency ablation
RTK	Receptor tyrosine kinase
TACE	Transarterial chemoembolization
TCF	T-cell factor

## References

[1] Kumaravel J., Selvamani M., Elangovan D., Subramanian B. (2024). Exploring the Potential of Artificial Intelligence and Machine Learning for Cancer Diagnosis and Treatment: Recent Progress and Looking Ahead. Oral Oncol. Rep..

[2] Sudhakaran G. (2024). Artificial Intelligence in Reducing Delays and Enhancing Accuracy of Oral Cancer: Recent Affairs. EJMO.

[3] Veeraraghavan V.P., Minervini G., Russo D., Cicciù M., Ronsivalle V. (2024). Assessing Artificial Intelligence in Oral Cancer Diagnosis: A Systematic Review. J. Craniofac. Surg..

[4] Kim E., Viatour P. (2020). Hepatocellular carcinoma: Old friends and new tricks. Exp. Mol. Med..

[5] Sanyal A.J., Yoon S.K., Lencioni R. (2010). The Etiology of Hepatocellular Carcinoma and Consequences for Treatment. Oncologist.

[6] Chidambaranathan-Reghupaty S., Fisher P.B., Sarkar D. (2021). Hepatocellular carcinoma (HCC): Epidemiology, etiology and molecular classification. Adv. Cancer Res..

[7] Pandyarajan V., Govalan R., Yang J.D. (2021). Risk Factors and Biomarkers for Chronic Hepatitis B Associated Hepatocellular Carcinoma. Int. J. Mol. Sci..

[8] Chavda V., Zajac K.K., Gunn J.L., Balar P., Khadela A., Vaghela D., Soni S., Ashby C.R., Tiwari A.K. (2023). Ethnic differences in hepatocellular carcinoma prevalence and therapeutic outcomes. Cancer Rep..

[9] Xue Y., Ruan Y., Wang Y., Xiao P., Xu J. (2024). Signaling pathways in liver cancer: Pathogenesis and targeted therapy. Mol. Biomed..

[10] Aden D., Zaheer S., Sureka N., Trisal M., Chaurasia J.K., Zaheer S. (2025). Exploring immune checkpoint inhibitors: Focus on PD-1/PD-L1 axis and beyond. Pathol. Res. Pract..

[11] Wojtukiewicz M.Z., Rek M.M., Karpowicz K., Górska M., Polityńska B., Wojtukiewicz A.M., Moniuszko M., Radziwon P., Tucker S.C., Honn K.V. (2021). Inhibitors of immune checkpoints—PD-1, PD-L1, CTLA-4—New opportunities for cancer patients and a new challenge for internists and general practitioners. Cancer Metastasis Rev..

[12] Mandlik D.S., Mandlik S.K., Choudhary H.B. (2023). Immunotherapy for hepatocellular carcinoma: Current status and future perspectives. World J. Gastroenterol..

[13] Han S., Sung P.S., Park S.Y., Kim J.W., Hong H.P., Yoon J.-H., Chung D.J., Kwon J.H., Lim S., Kim J.H. (2024). Local ablation for hepatocellular carcinoma: 2024 expert consensus-based practical recommendation of the Korean Liver Cancer Association. J. Liver Cancer.

[14] Ho S.-Y., Liu P.-H., Hsu C.-Y., Huang Y.-H., Liao J.-I., Su C.-W., Hou M.-C., Huo T.-I. (2022). Radiofrequency Ablation versus Transarterial Chemoembolization for Hepatocellular Carcinoma within Milan Criteria: Prognostic Role of Tumor Burden Score. Cancers.

[15] Liu M., Wen Y. (2024). Point-of-care testing for early-stage liver cancer diagnosis and personalized medicine: Biomarkers, current technologies and perspectives. Heliyon.

[16] Yıldırım H.Ç., Kavgaci G., Chalabiyev E., Dizdar O. (2023). Advances in the Early Detection of Hepatobiliary Cancers. Cancers.

[17] Canbay A. (2021). Prevention of hepatocellular carcinoma and surveillance of high-risk patients. Hepatol. Forum.

[18] Yang J.D., Hainaut P., Gores G.J., Amadou A., Plymoth A., Roberts L.R. (2019). A global view of hepatocellular carcinoma: Trends, risk, prevention and management. Nat. Rev. Gastroenterol. Hepatol..

[19] Jabri A., Khan J., Taftafa B., Alsharif M., Mhannayeh A., Chinnappan R., Alzhrani A., Kazmi S., Mir M.S., Alsaud A.W. (2024). Bioengineered Organoids Offer New Possibilities for Liver Cancer Studies: A Review of Key Milestones and Challenges. Bioengineering.

[20] Fu J., Feng Y., Sun Y., Yi R., Tian J., Zhao W., Zhang C. (2024). A multi-drug concentration gradient mixing chip: A novel platform for high-throughput drug combination screening. Biosensors.

[21] Ioannis K., Theodoros P.V., Anna Z., George K.M. (2024). Towards Automation in Radiotherapy Planning: A Deep Learning Approach for the Delineation of Parotid Glands in Head and Neck Cancer. Bioengineering.

[22] Xi X., Li J.Q., Zhu Z.C., Zhao L., Wang H., Song C.W., Chen Y.N., Zhao Q., Yang J.J., Pei Y. (2024). A Comprehensive Review on Synergy of Multi-Modal Data and AI Technologies in Medical Diagnosis. Bioengineering.

[23] Gharahbagh A.A., Hajihashemi V., Machado J.J.M., Tavares J.M.R.S. (2024). Feature Extraction Based on Local Histogram with Unequal Bins and a Recurrent Neural Network for the Diagnosis of Kidney Diseases from CT Images. Bioengineering.

[24] Stolz M. (2024). The Revolution in Breast Cancer Diagnostics: From Visual Inspection of Histopathology Slides to Using Desktop Tissue Analysers for Automated Nanomechanical Profiling of Tumours. Bioengineering.

[25] Kong L., Huang M., Zhang L.F., Chan L.W.C. (2024). Enhancing Diagnostic Images to Improve the Performance of the Segment Anything Model in Medical Image Segmentation. Bioengineering.

[26] Lin C.Y., Wu J.C.H., Lu H.H.S., Kuan Y.M., Liu Y.C., Chang P.Y., Chen J.P., Lee O.K.S. (2024). Precision Identification of Locally Advanced Rectal Cancer in Denoised CT Scans Using Efficient Net and Voting System Algorithms. Bioengineering.

[27] Shamir S.B., Sasson A.L., Margolies R., Mendelson D.S. (2024). New Frontiers in Breast Cancer Imaging: The Rise of AI. Bioengineering.

[28] AlMohimeed A., Shehata M., El-Rashidy N., Saleh H. (2024). ViT-PSO-SVM: Cervical Cancer Predication Based on Integrating Vision Transformer with Particle Swarm Optimization and Support Vector Machine. Bioengineering.

[29] Zhang R., Liu Z.R., Zhu C.Y., Cai H., Yin K., Zhong F., Liu L. (2024). Constructing a Clinical Patient Similarity Network of Gastric Cancer. Bioengineering.

[30] Alsulami A.A., Albarakati A., AL-Ghamdi A.A., Ragab M. (2024). Identification of Anomalies in Lung and Colon Cancer Using Computer Vision-Based Swin Transformer with Ensemble Model on Histopathological Images. Bioengineering.

[31] Jeong W.S., Baek C.H., Lee D.Y., Song S.Y., Na J.B., Hidayat M.S., Kim G.W., Kim D.H. (2024). The Classification of Metastatic Spine Cancer and Spinal Compression Fractures by Using CNN and SVM Techniques. Bioengineering.

[32] Yang P.C., Huang C.W., Karmakar R., Mukundan A., Chen T.H., Chou C.K., Yang K.Y., Wang H.C. (2025). Precision Imaging for Early Detection of Esophageal Cancer. Bioengineering.

[33] Zaridis D.I., Pezoulas V.C., Mylona E., Kalantzopoulos C.N., Tachos S.S., Tsiknakis N., Papanikolaou N., Matsopoulos G.K., Tsiknakis M., Marias K. (2025). Simplatab: An Automated Machine Learning Framework for Radiomics-Based Bi-Parametric MRI Detection of Clinically Significant Prostate Cancer. Bioengineering.

[34] Zhang D., Zhao L.H., Wang B.J., Guo B., Guo A.H., Zhou Z.G. (2025). Integrated Machine Learning Algorithms-Enhanced Predication for Cervical Cancer from Mass Spectrometry-Based Proteomics Data. Bioengineering.

[35] Su C.C., Chou C.K., Mukundan A., Karmakar R., Sanbatcha B.F., Huang C.W., Weng W.C., Wang H.C. (2025). Capsule Endoscopy: Current Trends, Technological Advancements, and Future Perspectives in Gastrointestinal Diagnostics. Bioengineering.

[36] Soker A., Almagor M., Mai S., Garini Y. (2025). AI-Powered Spectral Imaging for Virtual Pathology Staining. Bioengineering.

[37] Moghadam S.D., Maris B., Mokhtari A., Daffara C., Fiorini P. (2025). OCT in Oncology and Precision Medicine: From Nanoparticles to Advanced Technologies and AI. Bioengineering.

